# Host–Biofilm Interactions

**DOI:** 10.3390/microorganisms10081641

**Published:** 2022-08-13

**Authors:** Yun Chen, Ilana Kolodkin-Gal

**Affiliations:** 1 State Key Laboratory of Rice Biology, Institute of Biotechnology, Zhejiang University, Hangzhou 310058, China; 2 Department of Plant Pathology and Microbiology, The Hebrew University of Jerusalem, Rehovot 7610001, Israel

In most natural, clinical and industrial settings, microorganisms preferentially exist in biofilms, structured communities that associate with biotic and abiotic surfaces. From a simplistic perspective, biofilms that associate with the biotic surface (except microbial surfaces) are host-associated [1]. These three-dimensional single-species or polymicrobial communities are embedded in self-produced polymers (primarily exopolysaccharides, proteins, and nucleic acids) that constitute the extracellular matrix and are frequently mineralized [2,3]. This differentiation process enables the intercellular exchange of metabolites, genetic material, and signaling molecules. Host-associated biofilms form a complex ecological system where the hosts contribute a dynamic supply of nutrients and signaling molecules, but also expose the bacteria associated with the tissue to their immune response. One example is in the intestinal tract, where members of the microbiota form higher-order structures (e.g., biofilms), as they are embedded in complex, self-produced polymeric matrices, adherent to each other and surfaces or interfaces, and have enhanced antimicrobial resistance, virulence, and quorum-sensing capacities [1].

An additional example is beneficial biofilms associated with the plant root, such as *Bacillus subtilis* and its clade members [4,5] where the adherent bacteria protect their hosts from fungal, bacterial, and viral pathogens [6,7]. In this collection, Qin et al. review the molecular circuits that regulate biofilm formation by *B. subtilis*, and their impact on the fitness of this bacterium is discussed [8]. As *B. subtilis* is frequently studied as a model for plant growth promoting species, mammalian probiotics, and biocontrol agents, unraveling the molecular basis of its biofilm formation cannot be underestimated.

Several genetically manipulatable bacteria are frequently used as models to study pathogenic biofilms. For example, *Pseudomonas aeruginosa*, *Staphylococcus aureus*, and *Enterococcus faecalis* are opportunistic pathogens. They are often studied due to their ability to cause persistent infections in immunocompromised patients, and to mediate device-related infections. Persistent biofilm infections are associated with increased tolerance and resistance to antibiotics and antibacterial agents. They have an increased frequency of persister cells—slow-growing cells that are naturally more tolerant to antibiotics that directly or indirectly target microbial cell division. In this collection, the capacity of different model systems to reflect the ecology of infection and, in particular, the interactions with biotic mineralized and organic surfaces was reviewed, focusing on *P. aeruginosa* and *E. faecalis* infections. The review highlights the importance of ex vivo systems to mimic the natural microenvironments of the infectious agents, providing proof of evidence for the failure of a single in vitro model system to predict microbial colonization [9].

While *E. faecalis* infections are one example of oral biofilm infection, dental caries is a well-established multifactorial biofilm-mediated chronic disease. The complex microenvironment within the oral cavity is affected by diet, hygiene, and the microbiota of the cariogenic ecosystem, which is a proficient producer of organic acids. The environment–phenotype relationship between the microbiota during homeostasis and dysbiosis with the oral cavity is reviewed here by Chen et al. in a review aimed at exploring the underlying mechanisms of transformation from commensal biofilm to cariogenic biofilm [10]. Among the discussed microbiota species are the dental pathogens *Streptococcus mutans* (*S. mutans*), *Bifidobacterium dentium*, and Scardovia species. Lactobacilli species seem to play a dual role being detected in caries-free subjects but also frequently associated with teeth lesions. The authors present an appealing case for polymicrobial synergy and dysbiosis in dental caries, their impact on the current therapeutic targets of cariogenic biofilm, and their control [10].

Biofilms associated with the skin reside in a complex microenvironment subject to host-derived peptide signaling. Therefore, studying the biofilm–host crosstalk may expose novel approaches for antimicrobial therapies in cosmetology and dermatology. Dysbiosis of the skin microbiome is a readout of the chemical and biological interactions between cutaneous bacteria and their response to human hormones. For example, *Staphylococcus (S.) epidermidis* can outcompete and suppress invasive *S. aureus*, an interaction that is expected to be fine-tuned by endocrine feedback. In this collection, Mart’yanov and colleagues study the impact of the human hormone norepinephrine (NE) on mono-species and dual-species biofilms of *S. aureus* and *S. epidermidis.* The authors expose the potential roles of NE in multispecies skin-associated communities and suggest that NE has a primary role as a regulator of biofilm growth and maturation and that biofilms of both species are more sensitive to NE in anaerobic conditions. This study also indicates that the suppression of *S. aureus* by *S. epidermidis* is facilitated by NE in dual-species biofilms [11].

While catecholamines (such as NE) are the most studied hormones as bacterial effectors compared with other hormones, they are poorly investigated. Natriuretic peptides (NUPs) are responsible for regulating cardiovascular homeostasis and osmoregulation. These peptides interact with bacteria as potential regulators of *P. aeruginosa*, which is associated with atypical skin infections and inhibits biofilm formation in a dose-dependent manner. Within this collection, the effect of two NUPs—ANP (atrial natriuretic peptide) and CNP (C-type natriuretic peptide)—was studied by Ovcharova end colleagues for *Cutibacterium (C.) acnes* and *S. aureus.* ANP was found to regulate dual-species biofilms of *C. acnes* and *S. epidermidis*. ANP allowed *C. acnes* to grow better in the presence of *S. epidermidis*. While antimicrobial peptides tend to target biofilm less efficiently than planktonic cells, ANP was more potent towards biofilms and less potent in planktonic cultures. This outcome represents a potential link to the ecological drivers coupling the development of the multispecies biofilm community in skin glands and follicles with the human regulatory systems [12].

An unconventional model for biofilm formation in the host is *Arcobacter butzleri*, an aerotolerant bacterium and a food pathogen associated with diarrhea in humans and animals. Salazar-Sánchez and colleagues examine the genetic basis for biofilm formation in *A. butzleri*, studied by characterizing the biofilm formation in mutants for genes associated with biofilm formation in related species. This study implied that the flagellum is a vital structure in the biofilm formation of *A. butzleri*. In most biofilm formers, as an organelle allowing motility, the flagellum has essential functions for accessing and mechanosensing surfaces. Still, the flagellum also functions as a part of the biofilm matrix. Salazar-Sánchez et al. also recognized enhanced adhesion in some flagellar mutants, indicating that, though important, a functional flagellum is not essential for biofilm formation in *A. butzleri*. Condition-dependent biofilm regulation was identified by (p)ppGpp synthase SpoT as *spoT* mutants all presented an increased ability to produce biofilms on polystyrene and stainless steel but had reduced biofilm-forming capacity on glass. An additional appealing candidate emerging from this study as a potential regulator of biofilm formation is LuxS, involved in synthesizing the autoinducer AI-2 [13].

An additional unconventional biofilm model studied in this collection by Lopes and colleagues is *Providencia stuartii*, a Gram-negative biofilm-forming opportunistic pathogen from the *Enterobacteriaceae* family. This bacterium is known for its ability to form biofilms and its multidrug resistance (MDR) phenotype. The carriage of an inducible chromosomally encoded AmpC β-lactamase allows *P. stuartii* to degrade penicillin and cephalosporin antibiotics efficiently. *P. stuartii* communicates and interacts (socializes) before the adhesion of cells onto surfaces. Outer membrane-embedded general-diffusion porins promote cell–cell interactions, forming floating communities of tightly packed cells. In this model, the formation of floating communities precedes surface-associated biofilms, suggesting that surface-associated assemblies are generated by the sedimentation of floating and tightly packed communities [14]. A detailed investigation of both floating cells and surface-associated communities revealed that the presence of urinary tract cues (urea, bicarbonate, and ammonia) has a role in their assemblage [14]. While Omp-Pst1 is the primary channel enabling urea penetration, bicarbonate, and ammonia into the periplasm, the porin Omp-Pst2 enables the resistance of community members to these toxic compounds. Interestingly floating communities and surface-attached biofilms responded to treatment differently, and their response was dependent on conditions mimicking the urinary tract.

Altogether, the works published in this Special Issue demonstrate that combining approaches from ecology, molecular biology, and infection biology is a powerful approach to study of clinically relevant biofilms. The information gathered from multiple biofilm models can generate applications that are of relevance to agriculture, medicine, and biotechnology.
